# Digital resources and interactive multimedia tools for breastfeeding promotion and support: a scoping review

**DOI:** 10.3389/fdgth.2026.1778405

**Published:** 2026-06-03

**Authors:** Ana Lizette Rojas-Rodríguez

**Affiliations:** Department of Health Sciences, Universidad Técnica Particular de Loja, Loja, Ecuador

**Keywords:** breastfeeding support, digital resources, health communication, mHealth, multimedia interventions, scoping review, social media

## Abstract

**Background:**

Breastfeeding is widely recognized as one of the most cost-effective public health interventions for improving maternal and child health outcomes. Nevertheless, breastfeeding indicators remain suboptimal worldwide despite strong international recommendations. In recent years, digital technologies have emerged as tools to support breastfeeding promotion, education, and continuity. However, the evidence on digital and multimedia breastfeeding interventions is heterogeneous and scattered across disciplines, limiting a comprehensive understanding of their scope and effectiveness. For the purposes of this review, “digital resources” refers broadly to digital platforms and technologies used to deliver breastfeeding-related information or support; “interactive multimedia tools” refers to resources integrating two or more media formats (e.g., text, audio, video, graphics) with user interaction; and “digital interventions” is used as an umbrella term encompassing both concepts.

**Objective:**

To systematically map and synthesize available evidence on digital resources and interactive multimedia tools used to promote and support breastfeeding, describing their characteristics, implementation contexts, target populations, reported outcomes, and limitations.

**Methods:**

A scoping review was conducted following the Arksey and O'Malley methodological framework and reported in accordance with PRISMA-ScR guidelines. The methodological approach was also aligned with selected recommendations from the Joanna Briggs Institute for scoping reviews. Searches were carried out in PubMed, the Virtual Health Library (VHL), Google Scholar, and the AI-powered tool Consensus between April 2023 and July 2024. Peer-reviewed publications in English and Spanish from the last 10 years addressing digital resources or interactive multimedia tools for breastfeeding promotion or support were included. Data were extracted and synthesized using a descriptive analytical approach.

**Results:**

A total of 23 studies published between 2019 and 2024 were included. The review identified a range of digital interventions, including social media platforms, mobile health (mHealth) applications, web-based resources, educational videos, telemedicine services, and multimedia materials. Most studies targeted pregnant women and breastfeeding mothers, often in contexts of social or economic vulnerability. Overall, digital interventions were associated with increased breastfeeding knowledge, improved maternal self-efficacy, enhanced access to information and peer support, and favorable perceptions. However, evidence regarding breastfeeding duration and exclusivity was inconsistent, and substantial variability was observed in intervention design, implementation strategies, and outcome measurement. Studies from both high-income countries (HICs) and low- and middle-income countries (LMICs) were identified, with social media campaigns and low-cost mobile approaches appearing particularly relevant in resource-constrained contexts.

**Conclusion:**

Digital resources and interactive multimedia tools represent promising complementary strategies for breastfeeding promotion and support. This scoping review highlights both the potential benefits and the heterogeneity of existing digital interventions, emphasizing the need for standardized, theory-informed, and context-sensitive approaches to strengthen evidence-based practice and future research in digital breastfeeding support.

## 1 Introduction

Breastfeeding is widely recognized as one of the most cost-effective public health interventions for improving maternal and child health outcomes across the life course. The World Health Organization (WHO) and UNICEF recommend early initiation of breastfeeding within the first hour of life, exclusive breastfeeding for the first 6 months, and continued breastfeeding up to two years of age or beyond, given its well-documented benefits for infant survival, immune development, cognitive outcomes, and maternal health (1, 2). Despite this strong evidence base, global breastfeeding indicators remain suboptimal, particularly with regard to exclusive breastfeeding and sustained breastfeeding practices over time (3, 4).

Breastfeeding practices are influenced by a complex interplay of determinants, including maternal knowledge, social support, health system practices, workplace conditions, and sociocultural norms. Increasingly, access to education and support delivered through digitally mediated communication strategies has emerged as an additional and relevant determinant (4, 5). In recent years, the rapid expansion of digital technologies has created both new opportunities and challenges for breastfeeding promotion and support. Social media platforms, mobile health (mHealth) applications, websites, virtual peer-support communities, podcasts, videos, and other multimedia resources are now widely used by mothers to seek information, emotional support, and practical guidance related to breastfeeding (6).

Digital resources have the potential to address several traditional barriers to breastfeeding support. In particular, they may expand access to information and support beyond conventional clinical settings by providing education and social support through mobile and digital platforms (7). These tools can help overcome barriers such as limited access to trained professionals, time constraints, and care in geographically isolated or hard-to-reach areas by enabling remote support when in-person contact is not feasible. Evidence from remotely delivered breastfeeding support interventions suggests a reduced risk of discontinuing exclusive breastfeeding, indicating that these strategies may help mitigate logistical and contextual barriers to effective support (8).

However, digital environments are not inherently neutral or risk-free. The quality, accuracy, and underlying commercial interests of breastfeeding-related digital content vary considerably, and exposure to misinformation or the promotion of breast milk substitutes disguised as educational content remains a significant public health concern (9).

Emerging evidence indicates that digital and multimedia interventions may positively influence breastfeeding-related knowledge, maternal self-efficacy, and continuation, particularly when such interventions are culturally sensitive, sustained over time, and integrated within health systems (10).

For conceptual clarity, this review distinguishes between “digital resources,” defined as digital platforms or technologies used to provide breastfeeding-related information or support, and “interactive multimedia tools,” defined as resources that combine multiple media formats with some degree of user interaction. Given that the literature frequently uses these terms inconsistently, the broader term “digital interventions” is adopted throughout this manuscript to refer collectively to both categories.

Nevertheless, the available literature on digital interventions for breastfeeding promotion and support is characterized by substantial heterogeneity in study designs, types of digital platforms (e.g., social media, mobile applications, websites, telemedicine), target populations, and reported outcomes. Furthermore, this body of evidence spans multiple disciplines including public health, nursing, nutrition, communication, and digital health making it challenging to integrate findings and develop a comprehensive understanding of the field (10).

In this context, a scoping review represents a particularly appropriate methodological approach to systematically map the existing evidence, clarify key concepts, identify the range of digital resources and multimedia tools used to support breastfeeding, and highlight knowledge gaps requiring further research. Unlike systematic reviews focused on assessing the effectiveness of specific interventions, scoping reviews are especially suitable for emerging and complex fields such as digital health applied to breastfeeding, where the evidence is diverse, interdisciplinary, and continuously evolving (11, 12).

From a conceptual perspective, digital breastfeeding interventions can be understood through behavioral frameworks such as the Health Belief Model, which posits that health-related decisions are shaped by perceived benefits, perceived barriers, and self-efficacy (13). In this context, digital environments may play a critical role in modifying these determinants by improving access to information, enhancing perceived support, and strengthening maternal confidence in breastfeeding practices. Furthermore, digital tools may act as cues to action, facilitating engagement with breastfeeding-related information and reinforcing health-promoting behaviors.

In addition, this review is aligned with broader digital health frameworks, including the WHO Global Strategy on Digital Health 2020–2025, which emphasizes the role of digital technologies in strengthening health systems and promoting person-centered care. Together, these conceptual perspectives support the interpretation of how digital interventions may influence knowledge, perceived benefits, self-efficacy, and access to support, providing a robust framework for understanding the role of digital technologies in breastfeeding promotion (14).

Therefore, the aim of this scoping review is to map and synthesize the available evidence on digital resources and interactive multimedia tools used for breastfeeding promotion and support, describing their characteristics, implementation contexts, target populations, reported outcomes, and main limitations. By providing a structured overview of the current landscape, this review seeks to contribute a conceptual and empirical foundation to inform future research design, guide the development of evidence-based digital interventions, and support policy and practice decision-making aimed at strengthening breastfeeding promotion in the digital era.

## 2 Methods

### 2.1 Study design

A scoping review was conducted to map, describe, and synthesize the available evidence on the use of digital resources and interactive multimedia tools for breastfeeding promotion and support. This methodological approach was considered appropriate given the emerging, interdisciplinary, and heterogeneous nature of the field, as well as the diversity of study designs, digital platforms, and outcomes reported in the literature.

The review followed the methodological framework proposed by Arksey and O'Malley, later refined by Levac et al., and was reported in accordance with the PRISMA extension for scoping reviews (PRISMA-ScR), and further informed by recommendations from the Joanna Briggs Institute.

The review question was structured using the PCC (Population–Concept–Context) framework, where the population included pregnant and breastfeeding women, the concept referred to digital resources and interactive multimedia tools, and the context encompassed healthcare, community, and digital environments.

### 2.2 Search strategy

The literature search was conducted between April 2023 and July 2024. The following electronic databases were searched: PubMed, the Virtual Health Library (BVS), and Google Scholar. Additional databases such as Web of Science and Scopus were explored; however, after screening, no additional studies meeting the inclusion criteria were identified. These databases were screened using the same eligibility criteria applied to the primary databases.

Searches were conducted in both English and Spanish. Although search terms were developed primarily in English due to the predominance of scientific publications in this language, the search strategy was applied across both English and Spanish contexts, including searches in Spanish-indexed databases and the review of titles and abstracts in Spanish to identify potentially relevant studies. However, no additional Spanish-language studies were identified beyond those retrieved through English search terms.

Filters were applied to limit the search to studies published within the last 10 years, in English and Spanish, and involving human populations. Additional filters were applied when available depending on the database, such as study type and relevance. Duplicate records were identified and removed manually prior to the screening process.

Databases were selected to capture relevant literature in the fields of public health, nutrition, breastfeeding, digital technologies, and digital health. As a complementary strategy, the artificial intelligence tool Consensus was used to identify additional potentially relevant studies, applying filters for year of publication and study type. This tool was used as a complementary source and not as a primary database. To ensure full reproducibility, the complete search strategies for all databases, including full search strings, Boolean operators, and applied filters, are provided in Supplementary Table S1.

#### 2.2.1 Search terms

MeSH descriptors and free-text terms in English were used and combined with Boolean operators. Main terms included: Breastfeeding, Lactation, Health education, medical informatics applications, Mobile applications, Smartphone, Wearable electronic devices, Telemedicine, Social networks, Digital resources.

One of the search equations used was: (“Breastfeeding” [MeSH Terms] OR “Lactation” [MeSH Terms]) AND (“Health Education” [MeSH Terms] OR “Medical Informatics Applications” [MeSH Terms] OR “Mobile Applications” [MeSH Terms] OR “Smartphone” [MeSH Terms] OR “Wearable Electronic Devices” [MeSH Terms] OR “Telemedicine” [MeSH Terms] OR “Social Media” [MeSH Terms]).

This equation represents a complete example of the search strategy applied in PubMed.

Search strategies were adapted to the syntax and indexing systems of each database. A representative search equation for PubMed is provided in the manuscript, and the complete search strategies, including Boolean operators and applied filters for each database, are presented in Supplementary Table S1 to ensure transparency and reproducibility.

Google Scholar handling. For Google Scholar, due to the high volume of results and the lower specificity of available filters, only the first 10 pages of results (sorted by relevance) were reviewed, following common methodological recommendations for scoping reviews.

### 2.3 Eligibility criteria

Inclusion criteria comprised original research articles, systematic reviews, narrative reviews, scoping reviews, and meta-analyses addressing the use of digital resources, multimedia technologies, or interactive tools for the promotion, support, or strengthening of breastfeeding. Eligible studies were published in English or Spanish within the last 10 years.

Exclusion criteria included duplicate records, studies not related to breastfeeding or digital technologies, and publications such as editorials, opinion articles, monographs, theses, and grey literature.

The study selection process was conducted in sequential stages following standard scoping review methodology. The screening process was conducted in two stages: (1) title and abstract screening and (2) full-text review. Initially, all identified records (*n* = 760) were screened based on titles and abstracts to exclude clearly irrelevant studies. This initial screening step allowed for the rapid exclusion of studies not related to the research topic, substantially reducing the number of potentially eligible articles. Each stage of the screening process was conducted sequentially using predefined inclusion and exclusion criteria to ensure a consistent and transparent study selection process.

Records that appeared potentially relevant were subsequently retained for further evaluation. After applying the predefined inclusion and exclusion criteria at the title and abstract level, 100 articles were considered eligible for full-text assessment.

Full-text review enabled a more detailed evaluation of study relevance, resulting in the exclusion of articles that did not meet the eligibility criteria, including those not aligned with the study objectives or corresponding to editorial content, opinion pieces, theses, or other non-eligible publication types. Ultimately, 23 studies met all inclusion criteria and were included in this scoping review.

The entire selection process was systematically documented using a PRISMA-ScR flow diagram. All stages of screening, including title/abstract screening and full-text assessment, as well as study selection and data extraction, were conducted by a single reviewer. Although the involvement of at least two independent reviewers is recommended to reduce selection bias, this study was conducted by one reviewer due to feasibility constraints, which is acknowledged as a methodological limitation. Therefore, this methodological decision may increase the risk of selection bias and should be considered when interpreting the findings.

### 2.4 Search results

The database search identified a total of 760 records across all sources, including 570 from PubMed, 30 from the Virtual Health Library (BVS), 100 from Google Scholar, and 60 from the Consensus AI tool.

All identified records were initially screened based on titles and abstracts to exclude clearly irrelevant studies. This screening step resulted in the exclusion of a substantial number of records that were not related to breastfeeding or digital technologies.

Following title and abstract screening, 100 records were considered potentially eligible and were assessed in full text in full text according to the predefined inclusion and exclusion criteria.

After full-text evaluation, 77 articles were excluded for not meeting the eligibility criteria, including lack of relevance to the study objectives or being classified as editorials, opinion articles, theses, or other non-eligible publication types.

Ultimately, 23 studies met all inclusion criteria and were included in this scoping review. These included 9 studies identified through PubMed, 3 from the Virtual Health Library, 7 from Google Scholar, and 4 from the Consensus AI tool.

**Figure 1:**
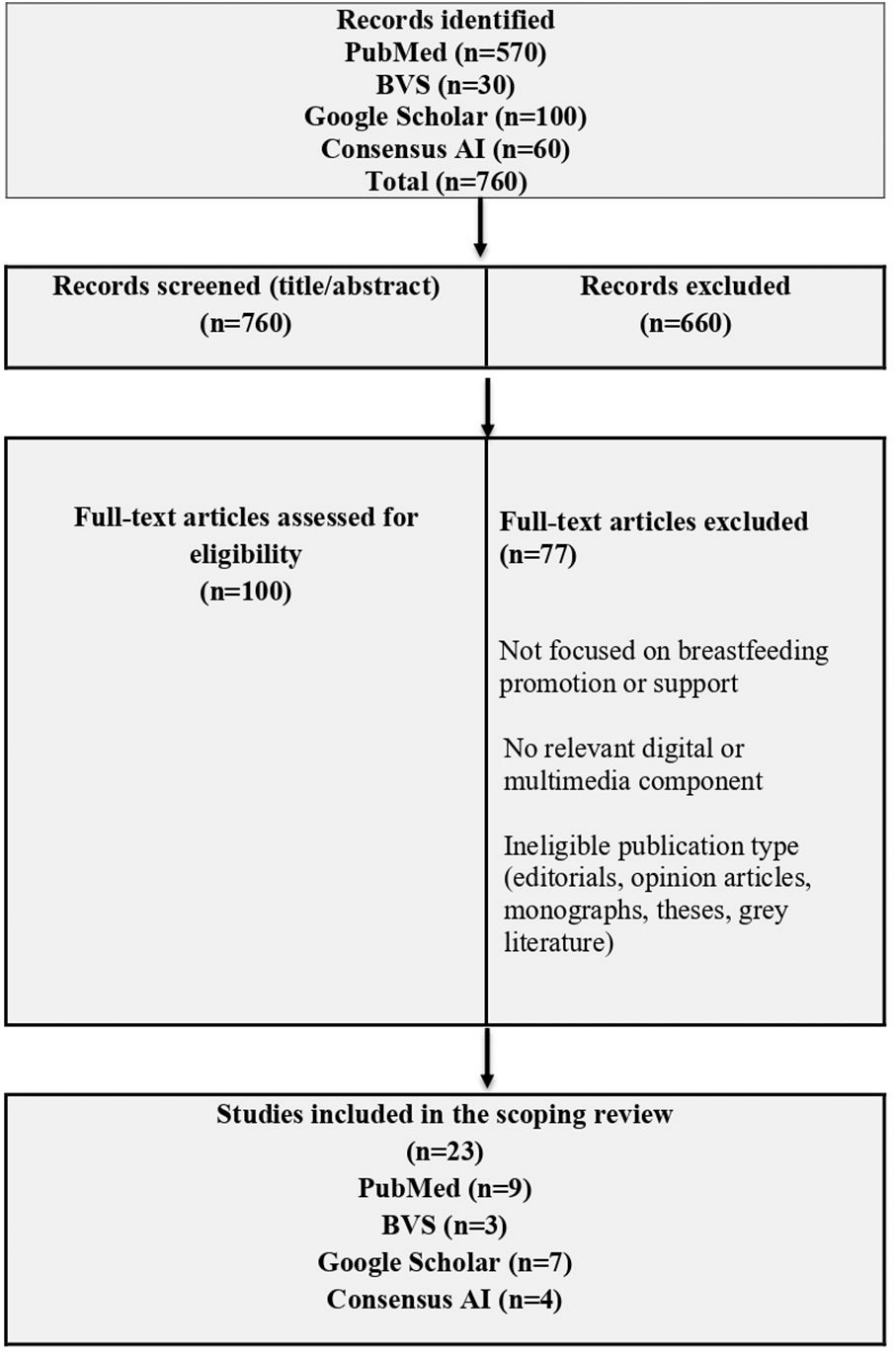
PRISMA-ScR flow diagram of study selection.

The study identification, screening, eligibility, and inclusion processes are summarized in the PRISMA-ScR flow diagram (Figure 1).

### 2.5 Data extraction and synthesis

Data were systematically extracted from each included study using a predefined data charting form. Extracted information included the type of digital or multimedia resource, digital platform employed, target population, implementation context, intervention characteristics, reported outcomes, and key findings.

The data extraction process followed a structured charting approach developed by the author and refined iteratively during the review to ensure consistency and completeness.

The results were synthesized using a descriptive and narrative approach, consistent with the objectives of a scoping review, to map the range, characteristics, and trends of digital and multimedia interventions supporting breastfeeding.

In accordance with scoping review methodology and the recommendations of the Joanna Briggs Institute, no formal methodological quality appraisal was conducted, as the purpose of this review was to map the available evidence rather than to assess the risk of bias or effectiveness of interventions.

## 3 Results

### 3.1 Study selection

The literature search conducted in PubMed, the Virtual Health Library (BVS), Google Scholar, and the artificial intelligence tool Consensus identified a total number of 760 records. After removal of duplicates and application of the predefined inclusion and exclusion criteria, 23 studies were included in this scoping review. The identification, screening, and selection process is summarized in the PRISMA-ScR flow diagram.

### 3.2 General characteristics of included studies

The 23 included studies, published between 2019 and 2024, originated primarily from the United States, Europe, Africa, and Asia, reflecting broad geographic diversity. Methodologically, the studies comprised systematic reviews, observational studies, randomized and non-randomized controlled trials, mixed-methods studies, content analyses, and evaluations of digital breastfeeding promotion campaigns.

Most studies focused on pregnant women, breastfeeding mothers, and postpartum women. Some also included pregnant adolescents, health professionals, and specific community-based populations, particularly in settings characterized by social or economic vulnerability. To improve interpretive depth, the included studies were also considered according to implementation setting, particularly distinguishing studies conducted in high-income countries from those developed in low- and middle-income countries, as this contextual variation may influence intervention design, feasibility, and reach.

When analyzed according to implementation context, differences emerged between studies conducted in high-income countries (HICs) and those developed in low- and middle-income countries (LMICs). While both contexts reported the use of digital tools, studies in LMICs more frequently focused on scalable, low-cost interventions aimed at expanding access and overcoming structural barriers to breastfeeding support, whereas studies in HICs more often described structured digital approaches integrated within health systems (15, 16) (Table 1).

**Table 1:** Characteristics of the studies included in the scoping review.

Country	Study design	Digital resource/platform	Target population	Main findings
Brazil	Narrative review	Social media, mobile apps, websites, videos	Not applicable	Mothersincreasingly use digital resources to seek breastfeeding information and support.
USA	Cross-sectional study	Facebook	WIC participants	Social mediainterventions facilitated effective breastfeeding promotion.
USA	Content analysis	Breastfeeding mobile apps	Mobile app users	Apps have potential but require improved educational content.
USA	Content analysis	Mobile applications	General users	Apps can support breastfeeding education and decision-making.
USA	Secondary analysis of RCT	Smartphone applications	Postpartum mothers	Technology-based interventions may increase breastfeeding rates.
USA	Mixed-methods	Twitter/X	General population	Strategic communication improves reach and engagement.
USA	Mixed-methods	Twitter/X	General population	Network structure influences breastfeeding message dissemination.
USA	Descriptive study	Educational film	Racially minoritized women	Digital media improves awareness of breastfeeding support.
Ghana	Descriptive-evaluative	Facebook, Twitter	General population	Social media campaigns effectively promoted breastfeeding.
Ghana	Campaign evaluation	Facebook, Twitter	General population	Paid ads increased exposure and engagement.
Saudi Arabia	Cross-sectional	Telemedicine	Breastfeeding mothers	High satisfaction with remote breastfeeding consultations.
USA	Systematic review	Web-based tools	Adolescent mothers	Digital tools positively influenced maternal outcomes.
UK	Observational study	Web-based resources	Parents	Digital resources improved confidence and bonding.
UK	Systematic review	Digital parental support	Parents	Integrated digital resources require institutional support.
Indonesia	Controlled trial	Educational videos, booklets	Pregnant women	Videos improved breastfeeding knowledge.
Indonesia	Meta-analysis	Smartphone-based education	Breastfeeding mothers	mHealth interventions improved exclusive breastfeeding.
USA	Observational study	Mobile applications	Pregnant and postpartum women	Apps offer broad informational support.
Indonesia	Non-RCT	Educational videos	Pregnant women	Videos were more effective than printed materials.
USA	Randomized trial	Educational video	Low-income women	Video alone insufficient for sustained impact.
UK	Systematic review	Social media	Breastfeeding mothers	Social media enhanced support and attitudes.
South Africa	Cluster RCT	Mobile video intervention	Pregnant women	Mobile videos improved breastfeeding education reach.
UK	Systematic review	Social media	Breastfeeding mothers	Online communities supported breastfeeding practices.

### 3.3 Types of digital and multimedia resources identified

The analysis identified a wide range of digital resources and multimedia tools used to promote and support breastfeeding across diverse contexts. The most frequently reported resources included social media platforms, mobile health (mHealth) applications, web-based platforms, educational videos and audiovisual campaigns, telemedicine services, and interactive multimedia materials.

Overall, social media platforms and mobile applications emerged as the most described tools. These resources were primarily used for disseminating breastfeeding-related information, facilitating peer support, and supporting community-based breastfeeding promotion initiatives.

Beyond the identification of digital tools, the findings reveal differences in the nature and function of digital interventions for breastfeeding promotion. Social media platforms appear to be predominantly aligned with community-based and peer-support interventions, prioritizing interaction, engagement, and rapid dissemination of information. In contrast, mobile applications and web-based platforms are more frequently associated with structured, education-oriented and behavior change interventions, incorporating functionalities such as monitoring, reminders, and personalized guidance. Telemedicine services, although less frequently reported, represent a distinct intervention modality focused on remote clinical support and direct interaction with healthcare professionals.

These differences suggest that digital breastfeeding interventions are not homogeneous, but rather reflect varying levels of complexity, intentionality, and integration within health systems. Furthermore, these variations may be influenced by the implementation context, as resource-constrained settings tend to prioritize more accessible and scalable strategies, whereas contexts with greater institutional capacity develop more structured and integrated interventions. Overall, these findings provide a deeper understanding of how the type of digital resource is linked to the purpose of the intervention and its implementation context (7, 16, 29).

#### 3.3.1 Digital platforms

Among the digital platforms reported, Facebook and Twitter/X were the most frequently used, particularly in studies examining health promotion campaigns, social network dynamics, and the dissemination of public health messages related to breastfeeding. These platforms were commonly leveraged to increase reach, foster interaction, and amplify evidence-based messages.

Mobile applications were mainly employed to provide structured educational content, breastfeeding tracking functions, reminder systems, problem-solving guidance for common breastfeeding challenges, and, in some cases, personalized support. Several studies also described the use of educational videos, either as standalone interventions or integrated into prenatal education programs, community-based initiatives, or broader digital health communication strategies (Table 2).

**Table 2:** Main outcomes associated with digital and multimedia breastfeeding interventions across included studies.

Outcome domain	Description	Representative studies
Breastfeeding knowledge	Improved understanding of breastfeeding practices, benefits, and management through digital education tools.	Pratiwi et al. (21); Wu et al. (28); Sidhu et al. (20)
Initiation, duration and exclusivity	Higher likelihood of breastfeeding initiation, continuation, or exclusive breastfeeding, particularly when interventions were integrated into broader support programs.	Harding et al. (16, 17); Adam et al. (25)
Breastfeeding self-efficacy	Increased maternal confidence and perceived ability to initiate and sustain breastfeeding.	Griffin et al. (22)
Social support	Enhanced peer-to-peer or professional support through social media platforms and digital communities.	Schindler-Ruwisch et al. (7); Orchard and Nicholls (10)
User satisfaction and engagement	Positive user experiences, acceptability, and engagement with digital tools.	AlHreashy et al. (27); Moukarzel et al. (18)

### 3.4 Methodological designs and target populations

The included studies exhibited substantial methodological heterogeneity. Identified designs comprised systematic and integrative reviews, randomized and non-randomized controlled trials, observational studies, content analyses, mixed-methods studies, and evaluations of digital health campaigns.

Regarding target populations, most studies focused on pregnant women, breastfeeding mothers, and postpartum women. However, several investigations specifically included first-time mothers, women from low-income settings, pregnant adolescents, racially minoritized communities, and populations living in low- and middle-income countries. This diversity reflects a growing interest in leveraging digital resources as strategies to reduce inequities in access to breastfeeding information and support.

### 3.5 Outcomes reported by included studies

Across the reviewed literature, the use of digital resources and multimedia tools was generally associated with positive outcomes related to breastfeeding support. Reported outcomes included increased breastfeeding-related knowledge, improved maternal self-efficacy, enhanced access to information and social support, and favorable user perceptions of digital technologies as complementary tools to traditional breastfeeding support.

Nevertheless, findings were heterogeneous with respect to breastfeeding duration and exclusivity. Positive effects on these outcomes were more consistently observed in studies where digital interventions were integrated into broader health care, community-based, or educational support programs, rather than implemented as standalone strategies.

Beyond these general findings, the results suggest that the effectiveness of digital breastfeeding interventions varies according to the type and level of structuring of the intervention. Social media–based interventions were more frequently associated with improvements in knowledge dissemination, perceived social support, and user engagement, whereas mobile applications and web-based tools demonstrated stronger associations with maternal self-efficacy and behavior-related outcomes. Notably, more consistent effects on breastfeeding duration and exclusivity were observed in interventions that were integrated within broader health systems or community-based support strategies.

These patterns indicate that the impact of digital interventions is not determined solely by the technology employed, but rather by the degree of integration, continuity, and alignment with existing health services. Furthermore, contextual differences appear to influence outcomes, as interventions implemented in low- and middle-income settings tend to prioritize access and coverage, while those in high-income settings more frequently target measurable behavioral changes and intervention effectiveness.

### 3.6 Synthesis of findings

Overall, the findings of this scoping review indicate a growing body of literature documenting the use of digital resources and multimedia tools to promote and support breastfeeding. This body of evidence is characterized by wide diversity in intervention approaches, digital platforms, methodological designs, and implementation contexts.

The available evidence suggests that digital and multimedia tools hold meaningful potential as complementary strategies for breastfeeding support, particularly by expanding reach and accessibility. However, substantial variation persists in their design, implementation, and evaluation, underscoring the need for more standardized, theory-informed, and context-sensitive approaches in future research.

Overall, the findings of this scoping review reveal a growing body of literature documenting the use of digital resources and multimedia tools for the promotion and support of breastfeeding, characterized by notable heterogeneity in intervention approaches, digital platforms, methodological designs, and implementation contexts.

While the available evidence suggests that these tools have relevant potential as complementary strategies particularly by expanding reach, accessibility, and opportunities for engagement the results indicate that this potential is neither uniform nor automatic.

Beyond this general perspective, the synthesis indicates that digital breastfeeding interventions should be understood as complex, context-dependent strategies with differentiated mechanisms of action. Their effectiveness appears to be less determined by the type of technology employed and more by structural factors such as the level of integration within health systems, the continuity of the intervention, and its alignment with behavior change frameworks. In this regard, interventions that combine digital components with clinical, educational, or community-based support demonstrate more consistent and sustained outcomes compared to those implemented as isolated tools.

Furthermore, the findings suggest that the implementation context plays a determining role in shaping both the design and impact of digital interventions. In low- and middle-income settings, strategies tend to prioritize accessibility, scalability, and the reduction of structural barriers, whereas in high-income countries there is a greater tendency toward more structured, digitally integrated interventions with a stronger focus on measurable behavioral outcomes. These differences reflect underlying disparities in health system capacity, digital infrastructure, and levels of digital literacy, underscoring the need for context-sensitive intervention design.

Taken together, these findings highlight the need to advance toward more standardized, theoretically grounded, and rigorously evaluated digital interventions, while also incorporating key dimensions such as equity, digital literacy, and long-term sustainability. The strategic integration of these tools within health systems emerges as a central element for maximizing their impact on breastfeeding promotion.

## 4 Discussions

This scoping review mapped and synthesized the available evidence on the use of digital resources and interactive multimedia tools for breastfeeding promotion and support, highlighting a heterogeneous, multidisciplinary, and rapidly expanding research field. Identified studies covered a broad range of digital platforms, methodological designs, geographic settings, and target populations, confirming the complexity of the digital ecosystem applied to breastfeeding.

### 4.1 Overview of digital resources used

The available evidence indicates that mothers increasingly rely on social media, mobile applications, websites, educational videos, and telemedicine services as sources of information and breastfeeding support (7, 15). Platforms such as Facebook, Twitter, smartphone applications, and web-based tools were identified as the most frequently used resources across both high-income and low- and middle-income settings (7, 16).

This pattern suggests that digital environments are not merely complementary or alternative channels to traditional in-person support, but rather function as adaptive support systems whose role varies depending on contextual constraints and access to formal healthcare services. In this regard, digital tools may compensate for structural barriers such as geographic limitations, time constraints, and restricted availability of specialized care.

### 4.2 Social media as spaces for dissemination, support, and engagement

The findings indicate that social media platforms function as effective tools for disseminating health-related messages, facilitating the development of virtual communities, and strengthening social support among breastfeeding mothers (15, 17). Campaigns implemented on Facebook and Twitter, particularly in contexts such as Ghana, demonstrated a strong capacity to reach large audiences, generate interaction, and deliver messages aligned with breastfeeding promotion (16, 17). However, studies also show that impact varies by platform, sponsored content use, dissemination strategy, and digital literacy level of the target audience (16, 18). Twitter, for instance, showed lower reach compared with Facebook, underscoring the need for tailored strategies by digital channel. This pattern suggests that digital environments are not merely alternative information channels, but function as adaptive support systems whose role varies depending on contextual constraints and access to formal healthcare services.

### 4.3 Mobile applications and web-based tools

Mobile breastfeeding applications represent another central area of the analyzed literature. Content analysis studies found these apps may improve knowledge, self-efficacy, and maternal confidence, although there is substantial variability in content quality, theoretical grounding, and interactivity (19, 20). Meta-analyses and controlled trials suggest smartphone-based educational interventions can positively contribute to exclusive breastfeeding, particularly when they include structured educational elements and audiovisual content (21, 22). However, some studies also warn that isolated digital interventions such as a single exposure to an educational video may be insufficient to produce sustained changes in breastfeeding duration or exclusivity, particularly in vulnerable populations (23). These findings suggest that the effectiveness of app-based interventions depends not only on content availability but on their ability to support sustained behavioral engagement.

### 4.4 Audiovisual and multimedia resources

Educational videos, promotional films, and interactive multimedia materials were identified as relevant tools for prenatal and postnatal breastfeeding education. The literature consistently indicates that audiovisual formats are more engaging and easier to understand than traditional printed materials, thereby facilitating the transmission of key messages and enhancing maternal knowledge (24, 25). However, their effectiveness appears to be contingent upon their integration within broader, structured intervention strategies that incorporate professional support, ongoing reinforcement, and cultural adaptation (23, 26). This suggests that audiovisual tools alone are insufficient to produce sustained behavioral change and are most effective when embedded within comprehensive intervention frameworks rather than implemented as isolated educational resources.

### 4.5 Telemedicine and remote support

Telemedicine for breastfeeding support, although less represented in the literature, showed promising results in terms of maternal satisfaction and accessibility, especially in contexts where in-person lactation consultation is limited (27). These findings reinforce the potential of remote services as a complement to traditional care, particularly in post-pandemic scenarios.

### 4.6 Information quality and associated risks

A cross-cutting finding across studies is concern about content quality, accuracy, and regulation. Systematic reviews and observational studies warn that exposure to unvalidated or potentially misleading information may negatively influence maternal decisions (28). Several authors emphasize the need for professionally curated platforms, active involvement of health professionals, and alignment with evidence-based guidelines (29).

### 4.7 Implications for research and practice

Overall, findings suggest digital and multimedia resources do not replace in-person support, but can play a key complementary role in breastfeeding promotion and support. The literature emphasizes the importance of integrated, culturally sensitive, sustained interventions that are articulated with health systems (28, 29).

Recent evidence also indicates that the effectiveness of breastfeeding interventions is strongly influenced by key behavioral determinants, including maternal intention, self-efficacy, and perceived social support (5). In this regard, interventions that combine education, support, and counselling, particularly when delivered through multiple contacts across the antenatal and postnatal periods, tend to achieve more consistent improvements in exclusive breastfeeding outcomes (5). These findings reinforce the interpretation that digital resources should not be understood as isolated tools, but as delivery mechanisms that can enhance continuity, accessibility, and personalization of support when integrated within structured intervention models.

Research gaps were identified, particularly in low- and middle-income countries, in long-term outcome evaluation, and in analyzing interactions between digital literacy, equity, and breastfeeding outcomes. These gaps highlight the need for future studies with more robust and comparable approaches to designing, implementing, and evaluating digital interventions.

### 4.8 Implications for policy and practice

Findings from this scoping review have relevant implications for public policy, digital health intervention design, and clinical practice in breastfeeding. Evidence suggests that digital resources and multimedia tools can expand the reach of breastfeeding support, particularly where barriers exist to accessing specialized in-person services, provided these resources are integrated in a planned manner within health systems and aligned with evidence-based recommendations (28, 29). From a policy perspective, it is necessary to strengthen regulatory frameworks and institutional strategies that ensure quality, accuracy, and transparency of breastfeeding-related digital content, limiting misinformation and the influence of breast milk substitute marketing in digital environments (10, 15). Results also support investment in digital public health campaigns, particularly in high-penetration social media platforms such as Facebook, which have demonstrated the ability to generate engagement and population reach at low cost when strategically designed (16, 18). In clinical and community practice, digital resources should be understood as complementary tools to professional support, capable of reinforcing prenatal education, maternal self-efficacy, and ongoing postpartum support, but not as substitutes for human contact and specialized counseling (19, 22). Training health professionals in the critical and ethical use of digital technologies, as well as co-creating culturally appropriate, mother-centered content, emerges as a key element to maximize impact. Overall, the strategic integration of digital resources into breastfeeding promotion policies and practices represents an opportunity to strengthen more accessible, equitable, and sustainable support systems, provided it is accompanied by continuous evaluation, appropriate regulation, and a rights- and experience-based approach.

### 4.9 Limitations

This scoping review has several limitations. First, the search was restricted to publications in English and Spanish, which may have led to the exclusion of relevant studies published in other languages. In addition, documents not indexed in the consulted databases were not included, so some reports or evaluations may not have been considered. Moreover, in accordance with scoping review methodology, no formal quality assessment of the included studies was conducted, which limits the ability to evaluate the strength of the evidence. Additionally, although standard recommendations for scoping reviews suggest the involvement of two or more independent reviewers in the study selection process, this review relied on a single reviewer, which may introduce a potential risk of selection bias. Finally, the heterogeneity observed in study designs, digital platforms, target populations, and evaluated outcomes, as well as the predominance of short-term results, hinders direct comparison across studies and limits conclusions regarding the long-term impact of digital interventions on breastfeeding.

## 5 Conclusions

This scoping review synthesizes current evidence on the use of digital resources and interactive multimedia tools for breastfeeding promotion and support, positioning these approaches within the field of digital health and health communication oriented toward behavior change. The findings indicate that digital platforms such as social media, mobile applications, web-based resources, audiovisual content, and telemedicine services are being used to influence key behavioral determinants, including breastfeeding-related knowledge, maternal self-efficacy, perceived social norms, and access to social support.

From the perspective of Frontiers in Digital Health, the results suggest that the potential of digital interventions to support breastfeeding lies not solely in the technology itself, but in the ways these tools mediate communication and behavioral processes. Interventions characterized by higher levels of interactivity, reliable content, and cultural relevance appear to foster greater user engagement and better alignment with behavior change objectives, particularly when integrated into broader maternal and child health strategies.

Nevertheless, the review reveals substantial heterogeneity in intervention design, theoretical grounding, and behavioral outcome assessment, which limits understanding of the mechanisms through which digital interventions influence breastfeeding practices. In addition, important gaps remain in the evaluation of long-term effects, the incorporation of equity and digital literacy perspectives, and the critical analysis of the digital communication ecosystem, particularly regarding misinformation and the marketing of breast milk substitutes.

Overall, these findings indicate that digital and multimedia tools represent a relevant component of digital health strategies to support behavior change in breastfeeding. However, their effectiveness depends on intentional, theory-informed design and user-centered approaches. Future research should prioritize behaviorally oriented health communication frameworks, longitudinal evaluations, and the responsible integration of digital interventions within health systems to maximize their impact on the promotion of sustainable and equitable breastfeeding practices.

## References

[1] World Health Organization. WHO recommendations on breastfeeding and donor human milk [Internet]. Geneva: World Health Organization (2023). Available online at: https://www.who.int/ (Accessed March 20, 2026).

[2] United Nations Children’s Fund (UNICEF). Infant and Young Child Feeding: Breastfeeding and Donor Human Milk. New York: UNICEF (2023).

[3] Victora CG Bahl R Barros AJD França GVA Horton S Krasevec J. Breastfeeding in the 21st century: epidemiology, mechanisms, and lifelong effect. Lancet. (2016) 387(10017):475–90. 10.1016/S0140-6736(15)01024-7 26869575

[4] Rollins NC Bhandari N Hajeebhoy N Horton S Lutter CK Martines JC. Why invest, and what it will take to improve breastfeeding practices? Lancet. (2016) 387(10017):491–504. 10.1016/S0140-6736(15)01044-2 26869576

[5] Whittaker X Meedya S Capper T. Factors and interventions that positively influence breastfeeding rates at six months postpartum: an integrative literature review. Women Birth. (2025) S1871–S5192(25):101904. 10.1016/j.wombi.2025.101904 40199118

[6] Lupton D. Digital health technologies and health promotion: a critical perspective. Health Promot Int. (2015) 30(1):134–42. 10.1093/heapro/dau091 25320120

[7] Schindler-Ruwisch JM Roess A Chiang S. Breastfeeding social support in the age of mHealth: a content analysis. J Hum Lact. (2018) 34(3):543–55. 10.1177/0890334418773302 29787686

[8] Gavine A Marshall J Buchanan P Cameron J Leger A Ross S. Remote provision of breastfeeding support and education: systematic review and meta-analysis. Matern Child Nutr. (2022) 18(2):e13296. 10.1111/mcn.13296 34964542 PMC8932718

[9] Save the Children, Baby Milk Action, International Code Documentation Centre. Digital marketing of breastmilk substitutes: scoping review final report [Internet]. London: Save the Children; 2024. Available online at: https://www.babymilkaction.org/wp-content/uploads/2024/07/STCF_Digital-Marketing-of-BMS-Scoping-Review_Final-Report_Jul2024.pdf (Accessed December 29, 2025).

[10] Orchard LJ Nicholls W. A systematic review exploring the impact of social media on breastfeeding practices. Curr Psychol. (2022) 41:6107–23. 10.1007/s12144-020-01064-w

[11] Arksey H O’Malley L. Scoping studies: towards a methodological framework. Int J Soc Res Methodol. (2005) 8(1):19–32. 10.1080/1364557032000119616

[12] Tricco AC Lillie E Zarin W O’Brien KK Colquhoun H Levac D. PRISMA extension for scoping reviews (PRISMA-ScR): checklist and explanation. Ann Intern Med. (2018) 169(7):467–73. 10.7326/M18-0850 30178033

[13] Jones CL Jensen JD Scherr CL Brown NR Christy K Weaver J. The health belief model as an explanatory framework in health behavior research: recent developments. Health Educ Behav. (2021) 48(6):728–35. 10.1177/10901981211045707

[14] World Health Organization. Global Strategy on Digital Health 2020–2025. Geneva: World Health Organization (2021).

[15] Galvão DMPG Batoca Silva EM Marques Silva D. Use of new technologies and promotion of breastfeeding: integrative literature review. Rev Paul Pediatr. (2022) 40:e2020234. 10.1590/1984-0462/2022/40/2020234 PMC8431999 34495273

[16] Harding K Pérez-Escamilla R Carroll G Aryeetey R Lasisi O. Four dissemination pathways for a social media–based breastfeeding campaign: evaluation of the impact on key performance indicators. JMIR Nurs. (2019) 2(1):e14589. 10.2196/14589 34345773 PMC8293701

[17] Harding K Aryeetey R Carroll G Lasisi O Pérez-Escamilla R Young M. Breastfeed4Ghana: design and evaluation of an innovative social media campaign. Matern Child Nutr. (2020) 16(2):e12909. 10.1111/mcn.12909 31867865 PMC7083481

[18] Moukarzel S Rehm M Caduff A del Fresno M Pérez-Escamilla R Daly AJ. Real-time twitter interactions during world breastfeeding week: a case study and social network analysis. PLoS One. (2021) 16(3):e0249302. 10.1371/journal.pone.0249302 33780502 PMC8007060

[19] Dinour LM Pole A. Evaluation of breastfeeding app features: content analysis study. JMIR Pediatr Parent. (2022) 5(4):e37581. 10.2196/37581 36287596 PMC9647452

[20] Sidhu S Ma K Sadovnikova A. Features and educational content related to milk production in breastfeeding apps: content analysis informed by social cognitive theory. JMIR Pediatr Parent. (2019) 2(1):e12364. 10.2196/12364 31518317 PMC6715395

[21] Pratiwi R Atmaka DR Sutoyo DAR Mahmudiono T. Effectiveness of smartphone-based nutrition education interventions on exclusive breastfeeding practice: a meta-analysis. Nutr Amerta. (2023) 7(4):615–25. 10.20473/amnt.v7i4.2023.615-625

[22] Griffin LB López JD Ranney ML Macones G Cahill AG Lewkowitz AK. Effect of novel breastfeeding smartphone applications on breastfeeding rates. Breastfeed Med. (2021) 16(8):614–23. 10.1089/bfm.2021.001 33826418 PMC8380791

[23] Kellams AL Gurka KK Hornsby PP Drake E Conaway MR. A randomized trial of prenatal video education to improve breastfeeding among low-income women. Breastfeed Med. (2018) 13(10):666–73. 10.1089/bfm.2018.0115 30351169

[24] Puspitasari D Sunarsih T. The effectiveness of education video and booklet media for pregnant mothers’ knowledge on preparation of breastfeeding practice. In: Proceedings of the International Conference on Health and Medical Sciences (AHMS 2020) (Paris: Atlantis Press) (2021).

[25] Adam M Tomlinson M Le Roux I LeFevre AE McMahon SA Johnston J. The Philani MOVIE study: a cluster-randomized controlled trial of a mobile video entertainment-education intervention to promote exclusive breastfeeding in South Africa. BMC Health Serv Res. (2019) 19(1):211. 10.1186/s12913-019-4000-x 30940132 PMC6444854

[26] Blackman KCA Slama DS Pickering TA Russell A Valentine W Merchant MA. Evaluation of a breastfeeding promotion film among a racially minoritized sample. BMC Pregnancy Childbirth. (2022) 22(1):262. 10.1186/s12884-022-04607-0 35346106 PMC8962205

[27] AlHreashy FA AlObeid GA Elbashir BM Alshathry AS. Telemedicine breastfeeding consultation: the Saudi experience. Cureus. (2023) 15(9):e45392. 10.7759/cureus.45392 37854766 PMC10580215

[28] Wu JJY Ahmad N Samuel M Logan S Mattar CNZ. The influence of web-based tools on maternal and neonatal outcomes in pregnant adolescents or adolescent mothers: mixed methods systematic review. J Med Internet Res. (2021) 23(8):e26786. 10.2196/26786 34435961 PMC8430830

[29] Crossland N Thomson G Morán VH. Embedding supportive parenting resources into maternity and early years care pathways: a mixed methods evaluation. BMC Pregnancy Childbirth. (2019) 19(1):253. 10.1186/s12884-019-2388-2 31331285 PMC6647328

