# Supplementary material for: Digital resources and interactive multimedia tools for breastfeeding promotion and support: a scoping review
Source: Front Digit Health. 2026 Jun 3;8:1778405. doi: 10.3389/fdgth.2026.1778405 (PMC13272134; doi:10.3389/fdgth.2026.1778405)
Supplement: Supplementary file 1 [file Table1.pdf]

Supplementary Table 1. Search strategy and study selection process

Database	Search strategy	Total records	After abstract screening	After full-text review	Studies retained before deduplication
PubMed	("Breastfeeding"[MeSH] OR "Lactation"[MeSH] OR breastfeeding OR lactation) AND ("Health Education"[MeSH] OR "Medical Informatics Applications"[MeSH] OR "Mobile Applications"[MeSH] OR "Smartphone"[MeSH] OR "Telemedicine"[MeSH] OR "Social Media"[MeSH] OR "interactive multimedia tools" OR "digital resources")	570	31	22	18
Virtual Health Library (BVS)	(breastfeeding OR lactation) AND (health education OR mobile applications OR telemedicine OR social media OR digital resources OR interactive multimedia tools)	50	20	10	3
Google Scholar	breastfeeding AND (digital resources OR multimedia OR mobile applications OR telemedicine OR social media)	982	20	9	6

Consensus AI	Query: "digital and interactive multimedia tools for breastfeeding promotion and support"	60	30	10	6

***Note**: The number of studies retained per database corresponds to records after full-text review before duplicate removal. The final number of included studies after deduplication and eligibility assessment was 23. Search strategies were adapted to the syntax, indexing systems, and retrieval functionalities of each database. Controlled vocabulary (MeSH terms) was used in PubMed, while equivalent free-text terms were applied in other databases such as BVS and Google Scholar to ensure comprehensive retrieval of relevant studies.*
